# Current Practice in the Measurement and Management of Vitamin D Status in Elite Sport and Parasport

**DOI:** 10.1002/ejsc.12305

**Published:** 2025-04-22

**Authors:** Daniel J. Owens, Andreas M. Kasper, Graeme L. Close

**Affiliations:** ^1^ Liverpool John Moores University Research Institute for Sport and Exercise Science Liverpool UK; ^2^ Newcastle United Football Club Newcastle‐upon‐Tyne UK

**Keywords:** elite athletes, sports nutrition, supplementation, vitamin D deficiency

## Abstract

The field of vitamin D research in sport has stagnated, with a notable lack of new evidence challenging existing paradigms. Despite well‐documented consequences of vitamin D deficiency in athletes, gaps remain in understanding the feasibility of best practices for measuring and managing vitamin D status in elite sports. This survey aimed to define whether practitioners test athletes’ vitamin D status, the methods used, decision‐making regarding supplementation and sources of information on vitamin D. Seventy‐four stakeholders from 26 sports and parasports participated, representing athletes of eight ethnicities across the United States, United Kingdom, Europe, Australia and Asia. Financial and logistical constraints were significant barriers to effective vitamin D testing and management. Testing practices varied widely, with venous blood sampling being the most common method. Many practitioners were unaware of the specific analytical methods used. Supplementation decisions were typically collaborative, involving the sport science support team, but approaches and criteria varied considerably among practitioners. Most sourced information were from academic literature and consultations. These results are the first to characterise the perceptions and practices of practitioners in elite sport and parasport regarding vitamin D testing and supplementation. Despite extensive literature on the vitamin D status of athletes and its impact on performance, our findings indicate stagnation in innovative practices for optimising vitamin D status. Technological improvements to reduce testing costs and collaborative approaches between practitioners and researchers could facilitate knowledge delivery and improve practices.

**Summary**

- This survey of 74 stakeholders across 26 sports highlights wide variability in vitamin D testing methods, with venous blood sampling being most common but limited awareness of analytical details among practitioners.
- Financial and logistical constraints pose significant challenges to consistent vitamin D testing and management practices in elite sports.
- Supplementation strategies typically involve multidisciplinary input, though criteria and approaches vary widely across practitioners and sports disciplines.
- Findings reveal a stagnation in novel practices for optimising vitamin D status, suggesting that technological advancements to reduce costs could enhance practical feasibility and foster improved protocols.
- There is still a need to characterise ethnic differences in vitamin D metabolism and how this impacts vitamin D recommendations.

## 1 Introduction

The field of vitamin D research in sport has seen few innovations in recent years and a subsequent lack of new evidence to challenge the status quo. The functional consequences of vitamin D deficiency for athletes are well understood and have been reviewed elsewhere (Owens et al. 2018). Similarly, several trials have captured the vitamin D status of athletes (as determined by the sum of serum 25[OH]D_2_ and 25[OH]D_3_ concentration), which all demonstrate that athletes show vitamin D deficiency when exposure to sunlight and dietary or supplemental vitamin D intake are insufficient. For example, sports in which training and competition are predominantly performed indoors show a high prevalence of deficiency such as basketball and ballet (Stojanović et al. 2022; Wolman et al. 2013). Athletes residing in sun‐rich environments also exhibit high rates of deficiency due to avoidance of the sun during the day (Hamilton et al. 2010). By comparison, very little is known about the feasibility of application of best practice as it relates to the measurement and management of vitamin D status in elite sport.

Best practice in elite sport also follows a different approach to the general population, since the goal is to maximise the limits of human performance. Dietary intake and supplementation with vitamin D for the general population is advised as a blanket approach that should prevent deficiency for the majority of people. However, similar to most phenomena, vitamin D status shows inter‐individual variability. This is particularly evident across the calendar year, predominantly as a consequence of changes in sun exposure due to cloud coverage, the angle of the sun (solar zenith), skin colour, genetic variation and individual sun exposure and dietary behaviours (Chen et al. 2007). Such variability would suggest that a blanket approach to the management of vitamin D status in elite athletes is sub‐optimal. However, from a practical perspective, blanket vitamin D management can save time and money, both of which can be scarce resources for performance teams, highlighting just one potential challenge of translating research to practice and the unique constraints of elite sport.

The only approach to individualise the management of vitamin D status at present is to assess serum vitamin D concentration and determine an appropriate intervention. In clinical practice, the measurement of vitamin D status is typically only recommended when patients are high‐risk and symptomatic for vitamin D deficiency. However, waiting for an athlete to become symptomatic for vitamin D deficiency in elite sport is negligent and therefore prevention is critical. In this regard, testing athlete vitamin D status at high‐risk periods of the year permits an individualised approach to the management of vitamin D status. Similar to the individualised supplementation, there are likely to be barriers to testing vitamin D status at different points over the year, such as financial budget to pay for testing and logistical constraints, such as blood sampling opportunities and shipping samples for analysis, particularly in large teams of athletes.

Taken together, there is an opportunity for innovation in the management of vitamin D status and beyond. As research‐active practitioners, it is our view that improvements to practice will only be achieved by viewing the management of vitamin D status through a practitioner lens rather than focusing on further trials to capture vitamin D status and its association with physiological outcomes. Therefore, the primary aim of this research was to characterise the perceptions and practices of practitioners surrounding vitamin D measurement and supplementation in elite sport and parasport. The purpose of collecting such data from the field is to inform future practitioner‐engaged research and to help design testing and supplementation policies that are informed by research and the challenges faced by support staff operating at the coalface of elite sport.

## 2 Methods

### 2.1 Ethical Approval

This study was granted an ethical approval by the Liverpool John Moores University Research Ethics committee (UREC approval number: 21/SPS/064). Written informed consent was provided by the expert panel members and survey participants.

### 2.2 Participants

Expert panel: Following the systematic process of identifying potential expert participants described in Section 2.2, we contacted the prospective experts by their work email/work telephone, where this information was publicly available.

Survey respondents: Key stakeholders involved in the decision‐making process related to vitamin D testing and supplementation in elite sport were invited to participate in the survey. To define training and performance calibre of the athlete(s) supported by the survey respondents, the participant classification framework proposed by (McKay et al. 2022) was employed. In brief, this system uses training volume and performance metrics to classify a participant to one of the following: Tier 0: sedentary; Tier 1: recreationally active; Tier 2: trained/developmental; Tier 3: highly trained/national level; Tier 4: elite/international level or Tier 5: world class. For the purpose of this study, only Tier 4 and Tier 5 respondents were included.

The sample size was determined based on the principle of information power, which suggests that the adequacy of a sample is dependent on the richness of the data and its relevance to the study aims rather than a predetermined number (Braun and Clarke 2016). Data collection ceased when the research team determined that sufficient responses had been gathered to meaningfully address the research questions. This decision was informed by an ongoing assessment of data saturation, ensuring that additional responses were not yielding new insights relevant to the study’s objectives.

### 2.3 Survey Validation

To validate the survey, we used the Delphi method: a systematic interactive forecasting method, which relies on a panel of experts. Delphi is based on the principle that decisions from a structured group of individuals are more accurate than those from unstructured groups. The experts answer questionnaires in two or more rounds. Linstone (1985) suggests that the minimum suitable expert panel size is seven, which is a generally accepted guide when planning and conducting Delphi studies. Dropout rates of 20% and 30% between subsequent rounds of questioning is expected (Bardecki 1984), so a suitable sample size for recruitment of the expert panellist is *n* = 10 to account for dropouts. Defining who is ‘an expert’ is subject to debate and there are several methods for doing so (Mauksch et al. 2020). We acknowledge suggestions by Kuchinke (1997), which state that part of being an expert is high commitment to the domain of expertise, which is in line with the general emphasis on deliberate practice in expert learning (Ericsson et al. 1993). To define how close the experts are to the topic, we propose objective closeness that is, familiarity with the topic as a result of exploration by research (as proposed by Needham and de Loë 1990). An advantage of using experts who are highly interested in topics at stake in Delphi surveys is a high initial response rate and a low tendency to drop out (Hasson et al. 2000). Taking the aim of the proposed study into consideration, we defined an expert as someone with maximum factual knowledge of the topic of vitamin D and/or the provision of professional sports nutrition services to elite athletes. In the current study, 12 experts were initially contacted, with seven experts accepting and completing all scoring rounds. Experts were asked to review and score each question of the survey on its relevance to the relevant section aim(s) using a 0–10 point Likert scale. Scoring was determined as follows:

#### 2.3.1 Scoring Round 1

- Following analysis of the audit from all experts, questions were accepted for the final survey with an average of 70% acceptance or greater.
- Questions were rejected from the survey if they average 30% or below.
- If an expert scored a question 4, 5 or 6 out of 10, they were asked to provide an alternative question or an amendment to the current question to improve its strength in relation to the section aim.

#### 2.3.2 Scoring Round 2

- Amended questions were then sent out to experts for round two of the Delphi process. Only questions that receive a 70% average or higher were accepted for the final questionnaire.

The survey was split into 4 sections that were designed to address four aims related to the overall purpose of the study.

- *Section 1 Aim:* to gain context about the practitioner and the athletic population that they work with (sample demographic).
- *Section 2 Aim:* to identify whether the practitioner tests vitamin D status in their athletes. If yes, the section will then identify what methods the practitioner uses to assess vitamin D status and how/if they use this information to inform their practice (testing practices).
- *Section 3 Aim:* to identify how practitioners determine if their athletes are supplemented with vitamin D, who makes this decision and what form, dose and frequency of vitamin D supplementation is used (supplementation practices).
- *Section 4 Aim:* to gain perspective on where practitioners get their information about vitamin D, testing vitamin D status and vitamin D supplementation and why practitioners do or do not believe testing and/or supplementation with vitamin D is important (sources of information and barriers to implementation).

After round 2 of scoring, there was > 70% agreement for all questions, and therefore, they were included in the final survey used in the study.

### 2.4 Data Collection and Handling

Prospective respondents were invited to participate in the current survey via social media platforms and contacted by email, where this information was publicly available. Invitations also encouraged prospective respondents to share the invitation to participate within their network or institution to maximise recruitment coverage. Survey responses were collected via Jisc Online Surveys tool (Jisc. Bristol, UK) and data exported to Microsoft Excel (Version 16.7) for analysis. Figures were produced in GraphPad prism (v.9 GraphPad Software, Boston, Massachusetts USA). Figures are presented as response rate = absolute number of responses.

### 2.5 Thematic Analysis

We conducted an inductive thematic analysis of open‐ended survey responses following the six‐step approach outlined by Braun and Clarke (2006). First, we familiarised ourselves with the data through repeated reading and initial note‐taking. Next, we generated codes across the dataset, capturing key features of the data relevant to the research question. These codes were then collated into potential themes, which were refined and reviewed to ensure that they accurately reflected the dataset. Themes were defined and named to best represent the patterns identified and a final analysis was conducted to produce a coherent narrative supported by illustrative data extracts.

## 3 Results

### 3.1 Sample Demographic

A sample of 74 survey respondents was obtained, with representation from the United Kingdom (*n* = 48), United States (*n* = 15), Europe (*n* = 5), Australia (*n* = 5) and Asia (*n* = 1). Twenty‐six sports were represented in the sample (see Table 1). Of the sample, 69% of practitioners worked predominantly with male athletes, 11% worked with female athletes and 20% worked with both male and female athletes.

**TABLE 1:** Sports represented in the study sample.

American football	Para cycling
Athletics (track)	Para judo
Basketball	Para Nordic
Cycling (road)	Para swimming
Cycling (track)	Para wheelchair basketball
Field hockey	Rugby union
Gaelic	Rugby league
Golf	Ski and snowboard
Ice hockey	Sliding sports (bobsled, skeleton, luge)
Judo	Soccer
Multisport institution	Swimming
Motorsport	Tennis
Netball	Volleyball

Survey respondents were predominantly nutritionists or dieticians, with small representation from physiologists, S&C coaches, club doctors or medical team member, sports scientists, performance directors and performance managers (Table 2).

**TABLE 2:** Professional roles of survey respondents.

	Count (*n*)
Nutritionist	46
Dietician	18
Physiologist	1
Strength and conditioning coach	3
Club doctor/medical team member	3
Other:	3
Performance director	1
Performance manager	1
Sports scientist	1

Given the role of ethnicity in vitamin D status, we found that it is important to characterise the ethnicities of the athletes that survey respondents worked with. There was a diverse pool of ethnicities; however, the predominant response was White Caucasian (Table 3).

**TABLE 3:** Ethnicities of athletes represented in the survey sample.

	Count (*n*)
Asian	4
Arab	0
Black African	13
Of Black African descent (i.e., Afro–European, African and American)	7
Pacific Islander	5
Hispanic or Latino	1
Mediterranean	5
White Caucasian	60
I do not know	0
Mix of races	21

### 3.2 Testing Practices

**FIGURE 1:**
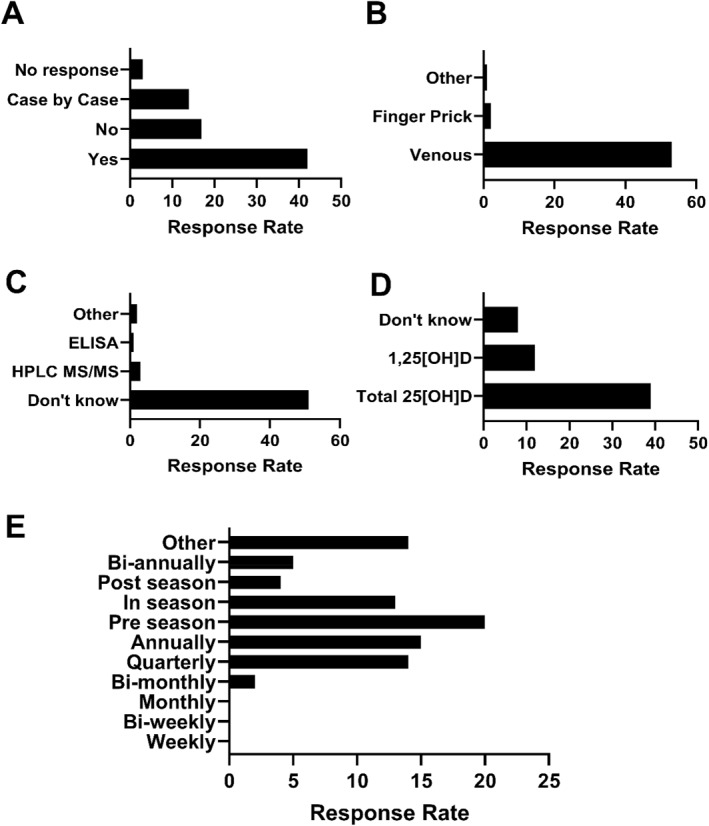
Vitamin D testing practices in elite sport. Panel (A) illustrates whether or not respondents in our sample test their athlete's vitamin D status. (B) Describes the method of blood collection used by the respondents. (C) Describes the method of chemical analysis used, if known, by respondents and (D) shows whether respondents measure total 25[OH]D, 1,25[OH]D or if they did not know what was measured. (E) Frequency of testing for vitamin D metabolites in athletes. Values are presented as the absolute number of responses for each survey question.

The large majority of those that did test their athlete's vitamin D status chose venous blood sampling (*n* = 53, 94%) with a small number opting for finger prick (capillary) blood sampling (*n* = 2, 3.5%), with the remaining participants not able to specify how samples were collected for analysis (Figure 1B). The analytical approach used to determine the vitamin D status by quantification of total 25[OH]D can significantly affect the results (Erdman et al. 2019; Snellman et al. 2010). Liquid Chromatography‐Tandem Mass Spectrometry (LC‐MS/MS) is the gold standard for the measurement of 25[OH]D_2_ and 25[OH]D_3_ and thus total 25[OH]D. Despite this, 89.5% (*n* = 51) of respondents who cited that they test their athlete's vitamin D status did not know what method was used to do so and reported sending their athletes samples to an external lab or hospital for analysis. Enzyme‐linked immunosorbent assay (ELISA), which can have cross‐reactivity with other vitamin D metabolites, potentially leading to over‐estimation or under‐estimation of 25[OH]D levels, was reported to be used by 1 respondent. Other methods, such as chemiluminescence immunoassay (CLIA), were reported to be used by 3.5% (*n* = 2) of respondents (Figure 1C).

The decision to supplement was largely made by a combination of the science, medicine and coaching team (*n* = 20, 30%), club doctor (*n* = 26, 38%) or nutritionist/dietician (*n* = 14, 21%) with only one respondent reporting that the physiotherapist and one other reporting that the physiologist was responsible. The frequency at which respondents reported testing their athlete's vitamin D status was highly heterogenous (Figure 1E). When asked to provide further information about testing frequency, themes that emerged related to a ‘seasonal approach to testing’, ‘testing coinciding with the competitive season’, ‘testing coinciding with baseline testing battery at the start of the competitive season’ and ‘testing when athletes are injured’. Case‐by‐case approaches are summarised in Table 4.

**TABLE 4:** Alternative and case‐by‐case approaches to vitamin D supplementation reported by respondents.

‘*If tested and supplemented, we follow‐up 8 weeks later.*’
‘*Sept/Jan/April or May depends on play offs.*’
‘*No defined structure.*’
‘*One‐off testing.*’
‘*Test July pre‐season and then ad hoc.*’
‘*If long term injury.*’
‘*Typically, we test early season (May), we follow‐up on athletes with insufficient levels anywhere from 6 to* *10 weeks later depending on competition/travel schedule.*’
‘*Jan, April, and usually one test in fall.*’
‘*Pre‐season (September/October) measures for all National* *Team athletes; selected tests mid‐season if pre‐season measures were low (< 50) OR symptoms possibly related to low Vit D (e.g. fatigue) persist in season; post‐season measures for any National* *Team athletes (March/April).*’
‘*Generally, twice a year, October to see if intervention required for winter. Then again in Jan as follow up for those on intervention and March/April for those not on intervention as another check point.*’

**FIGURE 2:**
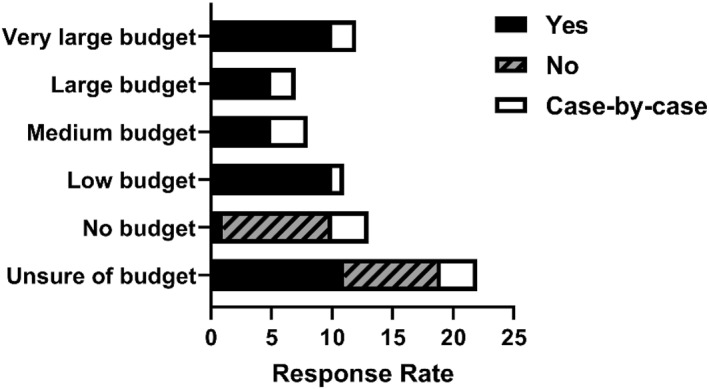
The relationship between budget and decision to test athlete vitamin D status. Budget was categorised as low (£1000; €1200; $1350 to £5000; $6000 and $6750), medium (£5000; €6000; $6750 to £10,000; €12,000 and $13,500), large (£10,000; €12,000; $13,500 to £20,000; €24,000 and $27,000) and very large (> £20,000; €24,000 and $27,000).

There was an apparent relationship between financial budget and the likelihood of practitioners testing their athlete's vitamin D status. Of those that answered that they do not test their athlete's vitamin D status (24%; Figure 1A), all responded having no budget or not knowing if there was a budget available. Of those respondents with some budget (low, medium, large or very large), all reported that they test all or at least some of their athlete's vitamin D status on a case‐by‐case basis (Figure 2).

### 3.3 Decision‐Making and Supplementation Practices

**FIGURE 3:**
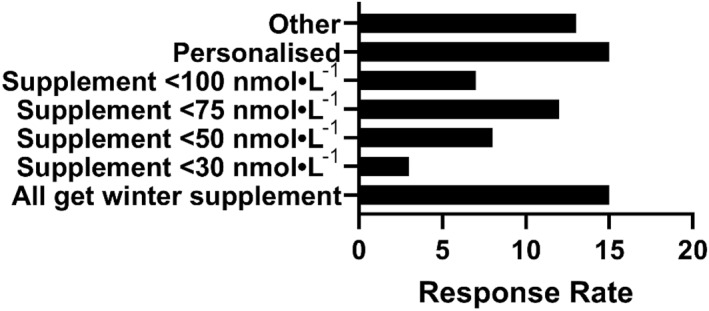
Strategies used by practitioners in elite sports to determine vitamin D supplementation for athletes.

When asked how the decision to supplement is made, a large proportion of respondents reported that all athletes get a winter supplement without any testing of vitamin D status (*n* = 15, 20%) and a similar proportion reported that all athletes receive a personalised approach (*n* = , 20%). Those that base supplementation upon 25[OH]D status used < 30 nmol·L^−1^ (*n* = 3, 4%), < 50 nmol·L^−1^ ^1^ (*n* = 8, 11%), < 75 nmol·L^−1^ (*n* = 12, 16%) and < 100 nmol·L^−1^ (*n* = 7, 10%) as the threshold for providing a supplement (Figure 3).

Eighteen percent (*n* = 13) of respondents selected ‘other.’ Upon further exploration, these responses included strategies such as providing all athletes with vitamin D supplements if their levels were below < 40 nmol·L^−1^ (*n* = 1) or < 90 nmol·L^−1^ (*n* = 2). One respondent reported advising athletes on which supplement to use, whereas others indicated they were unsure or did not provide further information.

**FIGURE 4:**
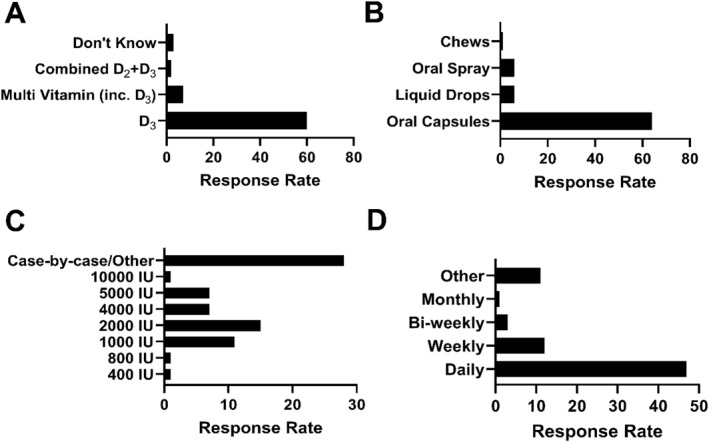
Vitamin D supplementation practices used by practitioners in elite sports. (A) Type of vitamin D provided to athletes, (B) form of vitamin D delivery, (C) quantity of vitamin D administered and (D) frequency of supplementation.

Vitamin D supplementation decisions were most commonly reported as a joint responsibility of the athlete support team (*n* = 34, 47%), whereas 44% (*n* = 32) of respondents indicated they were solely responsible. Additionally, 8% (*n* = 6) reported that the club doctor made the decision. Similar tothese decision‐making approaches, vitamin D supplementation practices varied, particularly in dosage and frequency (Figure 4C,D). However, the form of vitamin D and mode of delivery were most commonly vitamin D_3_ and in oral capsule form, respectively (Figure 4A,B).

Although a high proportion of respondents claimed that they used a case‐by‐case or unique approach (*n* = 28, 39%), few were able to clearly articulate what that approach was or whether there was a framework within which they operate (Table 5).

**TABLE 5:** Case‐by‐case approaches to vitamin D supplementation reported by respondents.

‘*We have two supplement protocols based on basal 25OHD. Also consider other lifestyle factors, previous bloods and ethnicity when categorising players*’
‘*We test and give anyone under 90 nmol·L^−^* ^*1*^’^a^
‘*We don't test but advise they use a batch tested vitamin D supplement.*’
‘*Everyone has winter vitamin D supplement to March.*’
‘*Vitamin D*3 *+ K*_2_ *soft gels are available for use by players at the club.*’
‘*We test and give anyone under 40 nmol·L^−^* ^*1*^ *a supplement. Even ones in the lower* *40s*–*50s get a lower maintenance dose.*’
‘*We test, supplement for those under 40 ng·mL^−^* ^*1*^ *and each athlete has an individual plan based on vit D level.*’

*Note:* 25[OH]*D* = 0.4 ng·mL^−1^.

^a^ 1 nmol·L^−1^.

### 3.4 Perceptions and Perceived Barriers Related to Testing and Supplementation With Vitamin D

The closed‐ended questions from this survey provided valuable data on testing, supplementation and decision‐making. However, the complex and nuanced nature of service provision in sports might be better understood through open‐ended survey responses. We asked practitioners to identify the biggest barriers, if any, to testing and managing athletes’ vitamin D status with supplementation. Several clear themes emerged from the data, which are summarised in Table 6.

**TABLE 6:** Perceived barriers to effective practice experienced by key stakeholders as determined by open‐ended question responses.

Illustrative extracts	Emergent theme
‘*Limited by budget constraints placed by funders of the organisation*’	Financial constraints for blood testing
‘*Logistics is a challenge especially for mid‐season and post season* *measurements. Game and practice schedules make it very difficult to find an appropriate day to draw blood*’	Logistical constraints for blood testing and athlete supplementation
‘*…it can be complex when determining an individual supplementation plan as some cases are not straight forward. E.g. black African athletes may have good adherence to supplementation but still present with suboptimal vitamin D in follow up blood tests. (Is free vitamin d a better option for these athletes?)*’	Lack of perceived consensus regarding testing and supplementation with or without reference to Black athletes
‘*[I*'*m] not sure if the team management would appreciate the importance of it to spend a portion of the budget on it*’	Lack of perceived benefit
‘*Adherence to daily supplement intake within athletes can also be challenging due to no noticeable benefit being seen from supplement as its impact is not directly ergogenic*’	Lack of compliance/adherence to supplementation

*Note:* Raw data include illustrative extracts from open‐ended question responses relevant to the theme.

### 3.5 Sources of Information on Vitamin D Supplementation and Testing Practices Used by Practitioners

The primary goal of collecting the data presented here is to inform future practitioner‐engaged research and to aid in designing testing and supplementation policies that are grounded in research and address the challenges faced by support staff in elite sports. We gathered information on where practitioners obtain their knowledge about vitamin D supplementation and testing practices to develop effective platforms for future knowledge transfer. Most practitioners reported using academic literature (*n* = 71, 97%), with a significant portion also consulting with academics (*n* = 46, 63%). Additionally, some practitioners referred to podcasts (*n* = 22, 30%), whereas a small percentage used social media (*n* = 3, 4%) and blog posts (*n* = 2, 3%) as sources of information.

## 4 Discussion

The primary aim of this research was to characterise the perceptions and practices of practitioners surrounding the vitamin D measurement and supplementation in elite sport and parasport. The purpose of collecting such data from the field is to inform future practitioner‐engaged research and to help design testing and supplementation policies that are informed by research and the challenges faced by support staff operating at the coalface of elite sport. Using a closed‐ended and open‐ended survey, we collected data from 73 practitioners spanning 26 different sports from the United Kingdom, United States, Europe, Australia and Asia. Following characterisation of the respondent demographic and the athletic population they work with, the survey aimed to address three main themes; to determine whether practitioners test vitamin D status in their athletes and if so, what analytical methods practitioner use to assess vitamin D status and how/if they use this information to inform their practice. Secondly, to identify who makes this decision to supplement the athlete and what form, dose and frequency of vitamin D supplementation is used. Finally, to gain perspective from where practitioners get their information about vitamin D, testing vitamin D status and vitamin D supplementation and why practitioners do or do not believe testing and/or supplementation with vitamin D is important.

Vitamin D deficiency in elite athletes has been widely reported, with results showing a decline in vitamin D status during the winter months. There are well‐established risk factors for vitamin D deficiency that are relevant to both the general population and athletes. Cloud cover, clothing and an indoor lifestyle (including indoor athletic training) all minimise UVB exposure and therefore limit the cutaneous synthesis of vitamin D (Chen et al. 2007). This decline in cutaneous synthesis is abetted by the fact that little vitamin D comes from foods, together leading to low total 25‐hydroxyvitamin D levels during the winter months in athletic populations. There are currently few solutions to accurately determine vitamin D status without direct assessment of vitamin D metabolites from a blood sample. When asked whether respondents tested the vitamin D status of their athletes, a quarter of our sample stated that they do not test vitamin D status (Figure 1). However, of those that did not test, 20% supplemented their athletes during the winter months. A clear barrier to implementation that was realised from our survey was financial budget as evidenced by the fact that all practitioners that did not test their athlete's vitamin D status, reported having no budget to do so or being unclear of the budget (Figure 2). Additionally, open‐ended survey responses indicated that budget and logistical challenges were important perceived barriers to implementing best practices (Table 6). Of those that did report the testing of vitamin D status, the majority did not know how the vitamin D status was tested, many of whom sent their samples to external laboratories for testing. This is a practical approach since no sports organisation is likely to have the means to accurately test vitamin D metabolites on site. However, knowing what method is used to test for 25[OH]D is crucially important. HPLC‐MS/MS is the gold standard for 25[OH]D testing, due to superior analytical specificity and sensitivity and wider dynamic range compared to other methods such as immunoassays (Snellman et al. 2010). Although global data on vitamin D testing procedures in private laboratories are scarce, it is reported that just 74 out of 364 US laboratories used LC‐MS/MS for 25[OH]D testing, yet 90% of routine 25[OH]D analyses are performed using automated immunoassays (Galior et al. 2018). Most automated immunoassays are limited by the inherently narrow dynamic range and specificity constraints of competitive immunoassays (Farrell et al. 2012; Holmes et al. 2013; Kocak et al. 2015). As a result, these assays often either underestimate or overestimate 25[OH]D concentrations, especially at the lower and higher ends of their measurement range, where accuracy is most crucial. The effect of analytical variability has previously been demonstrated through the re‐standardisation of 55,844 previously measured samples from 14 population studies, revealing significant overestimation or underestimation of 25(OH)D levels using most immunoassays (Cashman et al. 2016). Taken together, there is a high chance that most practitioners in elite sport and parasport are receiving unreliable and/or inaccurate 25[OH]D data for their athletes, yet 41% are using these data to inform the decision to supplement (Figure 3).

The timing of vitamin D status assessment is also important, particularly if it is to be used to inform the supplementation strategy. Findings from those with the budget to test their athletes indicate heterogeneity in testing frequency and period (Figure 1). When asked about ‘other’ or ‘case‐by‐case’ approaches, some practitioners describe a structure of testing over the winter months (when vitamin D levels are likely to be low due to a lack of sunlight exposure). For others, testing was ‘ad hoc’ or had no clear structure (Table 4). Unlike other micronutrients, vitamin D shows seasonal variation due to the dependency on sunlight exposure for a large proportion of vitamin D requirements, and thus a one‐time spot check for vitamin D status cannot provide insight across a competitive athletic season or competition cycle. However, one clear emergent theme from open‐ended survey responses is that practitioners frequently report not being able to find the budget to perform testing as they would like or have the logistical means to test at appropriate times. Therefore, viable solutions need to be developed to assist practitioners in making informed decisions as to when they should begin supplementation with vitamin D.

Practitioners expressed concern in open‐ended responses about what is the most appropriate vitamin D metabolite to measure, in some cases with reference to Black athletes (Table 6). In recent years, scientific literature has provided mixed messages about vitamin D testing. This stemmed from the emergence of an apparent Black athlete paradox, whereby Black athletes, despite presenting with consistently low total 25[OH]D, do not show the symptoms of vitamin D deficiency. For example, in a large cohort study of 604 athletes from 5 different ethnicities, there was no association between total 25[OH]D and bone mineral density (Allison et al. 2015). Black individuals have been reported to have higher 24 h calcium retention determined by subtracting calcium excretion through urine and faeces from dietary calcium intake and bone mineral density as determined using the z‐score from dual energy x‐ray absorptiometry (Weaver et al. 2008). Despite consistently lower total 25[OH]D, the absolute change in total 25[OH]D in response to supplementation (i.e., change relative to pre supplementation) is not different between White and Black subjects (Alzaman et al. 2016), which may indicate a lower requirement for vitamin D among Black individuals. Others have postulated that the bioavailable fraction of vitamin D is a better reference metabolite for vitamin D status (Powe et al. 2013), but this notion has since been disproved due to limitations in the assays used to determine bioavailable vitamin D (Henderson et al. 2016). Since the publication of the aforementioned research, little progress has been made in dissecting the aetiology of ethnic variance in vitamin D metabolism. The results presented here corroborate the need for clarity in individualised recommendations for vitamin D testing and supplementation, which first requires research efforts to improve our understanding of the vitamin D endocrine system across different ethnicities.

Finally, the results presented here provide evidence that effective educational materials and methods as well as behavioural interventions may be warranted for athletes with regards beliefs surrounding the importance of vitamin D and the motivation to take vitamin D supplements. When asked what the perceived barriers to implementation were for practitioners, two themes that emerged were a lack of perceived benefit and a lack of compliance/adherence to supplementation. As vitamin D is provided as a supplement, athletes appear to perceive it as an ergogenic aid akin to creatine or sodium bicarbonate. It is also evident that because it is a vitamin, some athletes believe the requirement should be met from food as evidenced by the adoption of the ‘food first’ as the preferred method for nutrition support within elite sport (Burke et al. 2019). Together these barriers may be attributable for the lack of adherence to supplementation reported by survey respondents here.

The results presented here highlight the diverse approaches to vitamin D testing and supplementation, along with various logistical challenges in knowledge delivery. Financial constraints significantly limit the effective management of vitamin D status, making the development of standardised guidelines for practitioners a crucial future objective. The authors, as research‐engaged practitioners, emphasise the continuing importance of translational research in the dynamic field of sports. Notably, a large portion of respondents seek information on vitamin D from academic journals, complemented by sources such as podcasts, blog posts, consultations with academics and social media.

In conclusion, these data are the first to characterise the perceptions and practices of a population of practitioners working in elite sport and parasport regarding vitamin D testing and supplementation. Despite a growing base of literature on the vitamin D status of athletes and the potential functional implications of vitamin D deficiency on athletic performance, results indicate stagnation in innovation regarding practice in the optimisation of vitamin D and athlete health and performance. Technological improvements to reduce the cost or need to test vitamin D status are needed, and practitioner–researcher co‐creation approaches could facilitate knowledge transfer and make step‐change improvements to practice.

## Conflicts of Interest

The authors declare no conflicts of interest.
